# A Case Report and Literature Review of Pheochromocytoma Without Tachycardia

**DOI:** 10.7759/cureus.57643

**Published:** 2024-04-05

**Authors:** Nikola Stojanovic, Emmanuel Ukenenye, Abdullah Khan, David Gunsburg

**Affiliations:** 1 Medicine, One Brooklyn Health-Brookdale University Hospital and Medical Center, Brooklyn, USA; 2 Internal Medicine, One Brooklyn Health-Brookdale University Hospital and Medical Center, Brooklyn, USA; 3 Cardiology, One Brooklyn Health-Brookdale University Hospital and Medical Center, Brooklyn, USA

**Keywords:** normetanephrine, metanephrines, palpitations, pheochromocytoma, tachycardia

## Abstract

This case report highlights a 47-year-old woman with an adrenal incidentaloma and a history of polysubstance abuse, finally diagnosed with pheochromocytoma. Characterized by episodic hypertension, headaches, and palpitations, pheochromocytoma is a rare condition with potential complications like uncontrolled hypertension and heart failure. Remarkably, during her 28-day hospitalization, continuous monitoring revealed no instances of tachycardia or arrhythmias despite multiple symptomatic episodes. This finding aligns with reports that while 50-70% of symptomatic pheochromocytoma patients experience palpitations, only about 20% exhibit detectable tachycardia or arrhythmias. This discrepancy suggests varied individual cardiovascular responses to catecholamine surges, possibly due to differences in catecholamine inactivation rates and receptor sensitivity. This case underscores the complexity of pheochromocytoma symptoms and highlights the need for personalized diagnostic and management strategies. Furthermore, it points to a significant gap in understanding the correlation between palpitations and arrhythmia in pheochromocytoma, indicating a critical area for future research.

## Introduction

In 1886, Felix Fränkel was credited with presenting the first case of pheochromocytoma. In that case, the patient was an 18-year-old woman who had both bilateral adrenal sarcoma and angiosarcoma. The onset of this woman’s illness included three episodes of sudden-onset palpitations with strong pulses, anxiety, headache, dizziness, vomiting, constipation, and progressively worsening weakness. She passed away on December 11, 1884, 10 days after admission [1].

Pheochromocytoma is a rare tumor that affects an estimated 0.8 per 100,000 people annually [2]. Fifty percent of patients with this tumor may lack symptoms and those that do typically experience paroxysmal clinical manifestations, which are sometimes misdiagnosed. The classic triad comprises tachycardia, diaphoresis, and headache episodes [3]. Intermittent or prolonged hypertension is the most frequent sign, followed by tachycardia, paroxysmal headaches, and widespread perspiration [4]. The most common cardiac rhythm abnormality in pheochromocytomas is sinus tachycardia, which typically manifests as palpitations [4]. Pheochromocytoma is rarely biochemically dormant and might be associated with more severe ventricular arrhythmias or conduction disturbances [5]. The treatment of symptomatic pheochromocytoma is essential because, if left untreated, it can progress to possibly fatal complications, like heart failure, arrhythmias, stroke, and malignant hypertension [6].

The median progression-free survival (PFS) of this neoplasm widely varies depending on whether the tumor is benign or malignant and also the tumor stage. Surgically extracted benign localized tumors can have an outstanding PFS with a significant number of patients without recurrence for many years. By contrast, for malignant, metastatic, or unresectable tumors, the PFS is usually around a couple of months, and a meta-analysis using tyrosine kinase inhibitor as a treatment modality noted a median duration of 8.9 months [7], while a retrospective study reported 4.1 months with sunitinib therapy [8]. Median overall survival (OS) and five-year overall survival also vary mainly based on the characteristics of the tumor and the management employed. Localized tumors generally have a more favorable OS and five-year overall survival than malignant or metastatic diseases as shown in a study for localized advanced pheochromocytoma, which revealed a one-year OS at 100% and five-year OS at 95%. The median OS in patients with recurrence was about 13 years [9]. 

Our case report portrays an unusual presentation of pheochromocytoma with paroxysms of hypertension, diaphoresis, and palpitation without tachycardia or arrhythmia during a month-long hospitalization and telemetry in the background of polysubstance abuse.

## Case presentation

This is a case of a 47-year-old African-American woman with a past medical history significant for adrenal incidentaloma diagnosed in 2022, single-vessel coronary artery disease (CAD) treated with a drug-eluting stent in 2013 (and on aspirin 81 mg), and polysubstance use (crack cocaine, tobacco, marijuana, and daily alcohol use) and received rehabilitation assistance, emphysema, controlled hypertension, type 2 diabetes mellitus, and hypercholesterolemia. The patient had a finding of an adrenal incidentaloma in 2022 that was biochemically inactive, but the patient was lost to follow-up.

The patient presented to the emergency room because of a five-day history of mid-sternal pressure-like chest pain radiating to the left breast associated with an episode of non-bilious non-bloody emesis. The patient’s initial vitals were blood pressure of 116/78 mmHg, heart rate (HR) of 65 bpm, respiratory rate (RR) of 18/min, and SatO_2_ of 100% on room air. She remained normotensive and without tachycardia.

The lab report indicated a slight elevation in high-sensitivity troponin levels to 17.3 ng/L, above the normal range of 5.0 to 11.8 ng/L. This level peaked further at 26.4 ng/L the next day, followed by a rapid decrease. The hemoglobin A1c was elevated at 9%. The rest of the laboratory workup, including complete blood count, comprehensive metabolic panel, urinalysis, pregnancy test, lipid panel, and thyroid function panel were within normal limits. Urine toxicology was positive for cocaine and cannabinoids.

The electrocardiogram (EKG) upon admission showed new T-wave inversions in leads II, aVF, V5, and V6, which were absent a month earlier. Given the patient's ongoing chest pain, nausea, history of hypertension, uncontrolled diabetes, and these recent changes in the EKG, the patient was hospitalized due to suspicions of unstable angina. A subsequent stress test confirmed stress-induced ischemia in the inferior lateral wall. However, coronary angiography was negative for obstructive CAD. The chest X-ray, the renal ultrasound, and abdominal X-ray were unremarkable. The echocardiogram showed a left ventricular ejection fraction (LVEF) of 65-70%, left ventricular hypertrophy, no regional wall motion abnormalities, and no valvular lesions.

The patient’s chest pain and nausea resolved, and she remained asymptomatic for a day and then started experiencing episodes of diaphoresis and elevated blood pressure, which went up to 220/135 mmHg and HR in the 70s range. During these episodes, she complained of palpitations and restlessness, but the review of the telemetry failed to show any arrhythmia or even tachycardia as her heart rate was stable in the 70s-80s range. 

**Figure 1:**
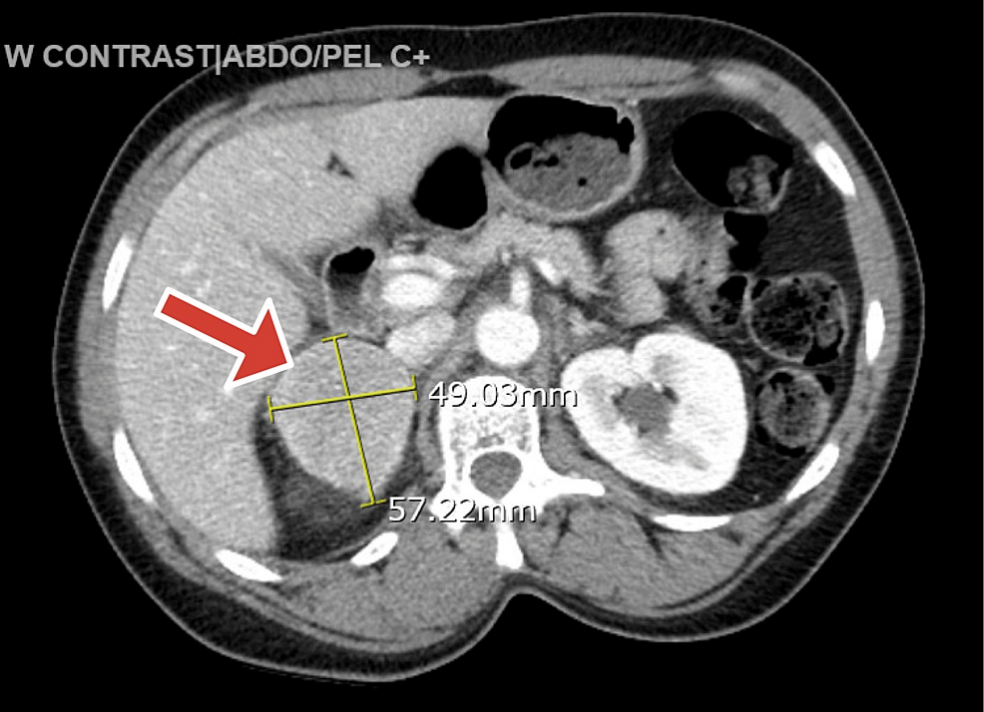
Computed tomography of the abdomen depicting the right-sided adrenal tumor

These episodes of elevated blood pressure were not controlled despite restarting the home regimen of antihypertensives, which included nifedipine 30 mg daily and losartan 50 mg daily, so the patient was started on lorazepam as needed for a possible alcohol withdrawal, and a nicotine patch was applied. With a known history of an adrenal tumor, the computed tomography (CT) of the abdomen with intravenous (IV) contrast was repeated and showed a 5.7 cm x 4.9 cm right adrenal mass (Hounsfield units (HU) ranging from 80 to 150 HU), as can be seen in Figure 1, and pheochromocytoma was suspected.

The pheochromocytoma workup came back positive: plasma normetanephrine was 3920 pg/ml (normal range: 0.0-218.9 pg/ml) and plasma metanephrines were 639.9 pg/ml (normal range: 0.0-88.0 pg/ml). Aldosterone, renin, an aldosterone-to-renin ratio, repeated morning cortisol, urine-free cortisol, parathyroid hormone, parathyroid-related peptide, serum calcium, and ionized calcium were within normal limits. 

The endocrinology team was consulted, and the thyroid ultrasound and an extensive workup for a mucosal neuroma in the search for multiple endocrine neoplasia type 2 (MEN 2) syndromes were unremarkable. The urology team was involved in the care, and a right-sided adrenalectomy was planned. Still, the patient needed to be pre-medicated with doxazocin 4 mg daily for at least seven days with a goal systolic blood pressure (SBP) in the range of 120-130 mmHg. After a week of adequate pre-medication, the SBP was slightly above the goal range, so the patient was started on atenolol 25 mg daily. Subsequently, the patient developed orthostatic hypotension, oral hydration was encouraged, and maintenance IV fluid was increased from 84 cc/hr to 100 cc/hr with immediate improvement in symptoms. A high-salt diet was started.

In the following days, the doxazosin dose was increased from 4 mg daily to 6 mg daily as per the anesthesiology recommendations, and the surgery was postponed for two days. Immediately preoperatively, the arterial and left internal jugular central lines were placed for close blood pressure monitoring, and the patient was medicated with IV phentolamine.

General anesthesia with propofol was achieved. During the surgery, the tumor was found to be adherent to the inferior vena cava, the liver, and the right renal hilum. The tumor was removed, and the sample was sent for histopathological evaluation. The immediate postoperative period was complicated by a period of hypotension that improved with aggressive IV fluid resuscitation and norepinephrine infusion. As a result, doxazosin and atenolol were discontinued and the patient was transferred to the surgical ICU for close monitoring. To decrease the chance of acute adrenal insufficiency, the patient was treated with hydrocortisone IV 100 mg, followed by 50 mg Q6hr and fludrocortisone 0.1 mg daily. 

The endocrinology team reassessed the patient the following morning and recommended discontinuing hydrocortisone and fludrocortisone. The patient’s blood pressure had improved, and further vasopressor support was not required. The patient's usual antihypertensives were restarted, and she was transferred to the regular medical floor. The further hospitalization course was uneventful, and the patient was discharged to a rehabilitation center with endocrinology, oncology, and urology outpatient follow-up.

The pathology report was positive for pheochromocytoma with lymphovascular invasion. The pathologic stage was assessed as pT2N0, and periadrenal adipose tissue was negative for tumor. Immunohistochemistry was positive for synaptophysin, chromogranin, GATA-3, and partially for S100. Rare focal positivity of Ki-67 with 1-2% proliferative index was found. The immunostains support the above diagnosis. The patient was followed up for six months, was asymptomatic, and continued to remain asymptomatic. The repeated CT abdomen and pelvis with IV contrast, hormonal profile, and regular labs were unremarkable. The patient will continue to be followed up.

## Discussion

What do we know about pheochromocytoma?

Pheochromocytoma, despite its rarity, is notoriously known for its intermittent sympathetic presentation, but over the past few decades, as high-resolution imaging techniques have become more widely used, more incidentalomas have been found. In addition, it has been reported that a higher percentage of all pheochromocytomas were identified during incidentaloma investigations [10].

An AI is clinically relevant as it has been noted to be associated with increased all-cause mortality in addition to being linked with a significantly high incidence of malignancy, impaired glucose tolerance, type 2 diabetes mellitus, hypertension, heart failure, dyslipidemia, peripheral vascular disease, renal disease, osteoporotic fractures, and chronic pulmonary disease [11]. These are the negative outcomes of continuous autonomous cortisol secretion seen in subclinical hypercortisolism. Much worse is the clinically silent pheochromocytomas, which correlate with quite high morbidity and mortality rates necessitating prompt treatment [12].

Clinical manifestations of pheochromocytoma

The spectrum of clinical manifestations of pheochromocytoma spans from asymptomatic as an incidental finding on imaging at the one end of the spectrum to the full-blown clinical triad of tachycardia, hypertension, and diaphoresis episodes. The symptoms of pheochromocytoma are present in half of the patients and are typically paroxysmal. The classic triad consists of episodic headache, sweating, and tachycardia [2].

Reflex bradycardia of vagal origin caused by the sinus node's inhibition has been seen during hypertensive bursts in a few pheochromocytoma cases [13]. The cardiovascular effects of paroxysmal catecholamine surge account for >50% of mortality in pheochromocytoma [14]. Most patients with pheochromocytoma do not have the three classic symptoms, and patients with primary hypertension might have paroxysmal symptoms [15]. Among the symptomatic patients, the frequency of sustained or paroxysmal hypertension is 85-95%, headache is 90%, and generalized sweating is 60-70% [16].

According to one study, the prevalence of palpitations was between 50% and 70% among symptomatic patients [5]. Despite more than half of symptomatic patients experiencing palpitations, there have been limited studies that have documented the prevalence of tachycardia in pheochromocytoma. In one case series of 106 patients, tachycardia (HR >100 beats/min) was noted in 14% and bradycardia (<60 beats/min) in 10% of the patients [17].

Incorporating our patient in the discussion

The patient in our case study remained asymptomatic for an extended period and successfully managed her high blood pressure with home medications. However, her sporadic use of crack cocaine, along with other potential triggering substances, could likely account for the onset of her symptoms. During her 28-day hospital stay with continuous heart rhythm monitoring, she experienced several instances of headache, sweating, high blood pressure, and occasional chest pain, but no arrhythmias or heart rate fluctuations beyond 90 beats per minute or below 50 beats per minute were observed. EKG during these episodes was negative for any new ischemic changes. In addition, the absence of an atrioventricular (AV) block and beta-blocker/non-dihydropyridine calcium channel blocker use rules out these instances as a cause for the lack of tachycardia.

A provisional conclusion of our discussion

The sudden surge of catecholamines might acutely increase the contractility of the myocardium, which might in part explain why some patients experience palpitations despite the lack of arrhythmia. The previous studies showed the lack of correlation between hemodynamic parameters, such as arterial blood pressure and heart rate, with the plasma catecholamine concentration, and it was attributed to varying responsiveness of the cardiovascular system to catecholamines. Several factors including the rate of catecholamine inactivation, by the uptake mechanism and enzymatic degradation, the extent to which catecholamines diffuse and reach target cells, the inherent reactivity of the vascular smooth muscle, and finally receptor number and sensitivity could explain varying responsiveness [18].

There is a lack of scientific evidence explaining this discrepancy between palpitations and arrhythmia prevalence among symptomatic pheochromocytoma patients. Possibly individualized precision medicine could provide further insights into why some patients experience palpitations.

## Conclusions

Pheochromocytoma, a rare condition, often presents with episodic hypertension, headaches, sweating, and sometimes tachycardia, leading to uncontrolled hypertension, heart failure, myocardial infarction, stroke, and atrial fibrillation. Our case report focused on a 47-year-old woman with a history of adrenal incidentaloma and polysubstance abuse, who experienced pheochromocytoma paroxysms. Surprisingly, despite multiple pheochromocytoma crises during her 28-day hospitalization, continuous telemetry monitoring showed no tachycardia or arrhythmias.

Notably, while palpitations are noted in 50-70% of symptomatic pheochromocytoma patients, only 20% exhibit detectable tachycardia or arrhythmias. This discrepancy suggests individual variations in cardiovascular responses to catecholamine surges, possibly influenced by factors, such as catecholamine inactivation rate and receptor sensitivity. This case underscores the need for personalized approaches in managing pheochromocytoma and highlights the importance of further research to understand the relationship between palpitations and arrhythmias in this condition.
